# The m^6^A regulatory network in plant stress adaptation: from mechanisms to breeding potential

**DOI:** 10.3389/fpls.2026.1940258

**Published:** 2026-10-05

**Authors:** Jun Zhang, Sainan Jiang, Jie Yang, Jingjing Zou, Wen Xing, Zeqing Li

**Affiliations:** 1 Key Laboratory of National Forestry and Grassland Administration on Osmanthus fragrans, Hubei University of Science and Technology, Xianning, China; 2 College of Landscape Architecture, Central South University of Forestry and Technology, Changsha, China; 3 School of Pharmacy, Hubei University of Science and Technology, Xianning, China; 4 School of Nuclear Technology and Chemistry & Biology, Hubei University of Science and Technology, Xianning, China

**Keywords:** N^6^-methyladenosine, plant stress response, mRNA stability, translational regulation, crop stress-resistance breeding

## Abstract

N^6^-methyladenosine (m^6^A) is the most abundant internal modification of eukaryotic mRNA and is dynamically regulated by three classes of proteins: writers, erasers, and readers. Writers deposit methylation marks, erasers confer reversibility, and readers translate these signals into specific regulatory outcomes, collectively modulating mRNA stability, translation efficiency, splicing, and polyadenylation, thereby influencing plant stress adaptation. Studies across diverse plant species have revealed both conserved principles and species-specific variations in this regulatory network. Technological advances, such as methylated RNA immunoprecipitation sequencing (MeRIP-seq) and nanopore direct RNA sequencing, have enabled transcriptome-wide m^6^A profiling. However, functional validation of m^6^A modification in most crops remains limited to a few model species and controlled environments, and their practical breeding potential requires systematic evaluation. This review systematically summarizes the functions of m^6^A writers, erasers, and readers in crop stress responses, synthesizes the regulatory patterns at the levels of mRNA stability, translation efficiency, and splicing and discusses m^6^A-based breeding strategies and their current bottlenecks, providing a theoretical reference for stress-resilient crop breeding. CRISPR/dCas13-based targeted modification systems, together with methyltransferase overexpression or demethylase knockout strategies, have demonstrated breeding potential in several crops, although field-level validation remains limited. Future research needs to prioritize how the writer-eraser-reader network achieves stress-specific regulation without causing pleiotropic developmental defects, how m^6^A pattern differs between single and combined stresses, and how natural variation in m^6^A regulatory gene can be exploited for breeding. On this basis, establishing causal links between specific m^6^A sites and agronomic phenotypes, while integrating multi-omics approaches and precision editing technologies, will be essential to translate epitranscriptomic knowledge into crop improvement.

## 1 Introduction

Global climate change has intensified the frequency and severity of abiotic stresses—drought, salinity, extreme temperatures, and flooding—posing a significant threat to agricultural productivity and food security. Without effective adaptation measures, crop yields are projected to decline by 7% to 23% by 2050 under the most extreme climate change scenarios (Arif et al., 2025). Enhancing crop stress tolerance is therefore a pressing challenge for sustainable agriculture. Plant responses to environmental stress involve multiple post-transcriptional regulatory mechanisms, including N^6^-methyladenosine (m^6^A) modification. In plants, several protein components regulating m^6^A modification have been identified, including writers, erasers, and readers. These proteins maintain the dynamic equilibrium of m^6^A modification and regulate various biological processes (Liang et al., 2020; Wang X. et al., 2021; Zhao et al., 2022; Shen, 2023; Yao et al., 2023; Ambrosino et al., 2024; Ramakrishnan et al., 2024; Zhang D. et al., 2024; Shao et al., 2025; Trösch, 2025). For example, m^6^A modification is involved in responses to heat, UV, and biotic stress (Meyer et al., 2015; Xiang et al., 2017; Scutenaire et al., 2018; Robinson et al., 2019; Martínez-Pérez et al., 2021; Tang et al., 2022; Tao et al., 2025), and the demethylases ALKBH9B and ALKBH10B participate in viral defense and flowering transition, respectively (Duan et al., 2017; Shoaib et al., 2021; Tang et al., 2021; Han R. et al., 2023). However, m^6^A reader genes, particularly those in the *YTH* family, show distinct expression patterns across species (Hu et al., 2019), suggesting that m^6^A regulatory mechanisms may vary among species. Although considerable knowledge has been gained from studies of the model plant Arabidopsis, the functions of m^6^A modification in the stress tolerance of major crops, as well as the conservation and diversity of these functions, remain largely unclear (**Figure 1**).

**Figure 1:**
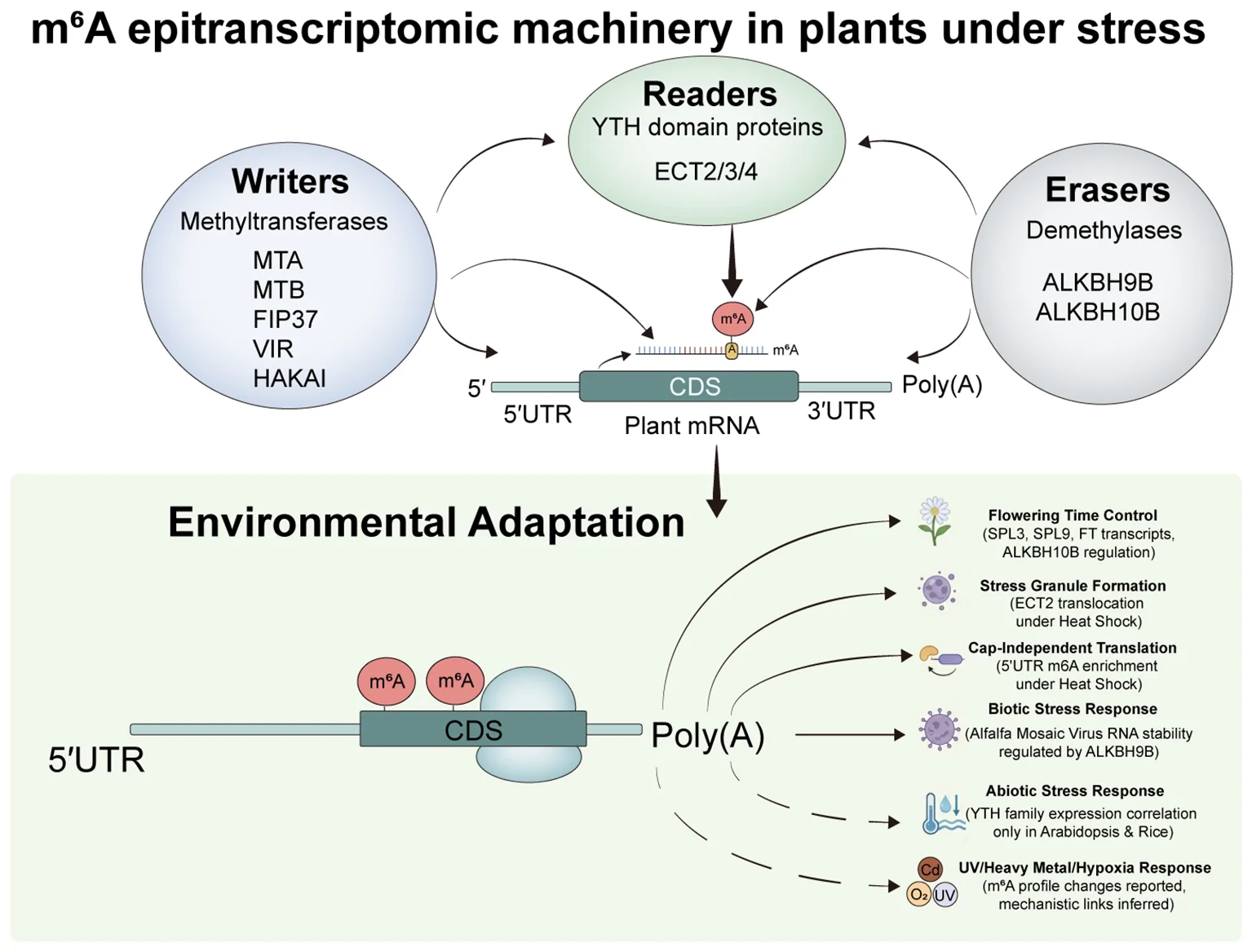
Overview of m^6^A modification and plant stress responses. The left panel summarizes experimentally validated m^6^A-mediated mechanisms (solid boxes/arrows); the right panel lists stress responses inferred primarily from expression correlations or m^6^A profiling, requiring functional validation (dashed boxes/arrows).

Unlike previous reviews that primarily focus on model plants or individual crop species, this review systematically surveys the functional roles of m^6^A writers, erasers, and readers in stress responses across diverse crop species. It synthesizes their regulatory patterns at the levels of mRNA stability and translation efficiency and discusses the conservation and species specificity of the regulatory mechanisms suggested by current evidence. We also summarize examples of m^6^A-based breeding strategies and discuss key challenges that constrain breeding applications. Finally, we highlight emerging technologies, such as single-cell epitranscriptomics, spatial transcriptomics, and multi-omics integration, for future m^6^A research in crops. This review aims to provide a theoretical reference for stress resistance breeding in food, cash, and horticultural crops. .

## 2 Methods

This narrative review synthesizes recent advances in m^6^A modification research in plant stress responses and breeding applications. We searched Web of Science and PubMed using the following keywords: “m^6^A”, “N^6^-methyladenosine”, “plant”, “stress”, “writer”, “methyltransferase”, “eraser”, “demethylase”, “reader”, and “YTH”. The search prioritized studies published between 2018 and 2026, a period during which plant m^6^A research expanded substantially. We also included foundational studies published before 2018 if they reported widely cited mechanisms or discoveries that remain relevant to the conceptual framework (e.g., the initial identification of MTA in Arabidopsis). Studies were selected for detailed discussion based on whether functional or mechanistic evidence was available (e.g., transgenic or mutant analyses); studies reporting only transcriptome-wide correlations were discussed as supporting evidence to indicate potential regulatory relationships that require further validation. All cited studies were evaluated for relevance to plant stress responses and breeding applications, with particular emphasis on food, cash, and horticultural crops.

## 3 Composition of the m^6^A regulatory network: writers, erasers and readers

The dynamic balance of m^6^A modification is maintained by three groups of proteins that work together: writers, erasers, and readers. Together, they form the m^6^A regulatory network. As reviewed by Yue et al., this network functions in Arabidopsis through the combined action of these three protein types, controlling mRNA stability and translation efficiency (Yue et al., 2019). Writers add methyl groups to specific sequence motifs, whereas erasers remove these groups, making the modification reversible. Readers recognise m^6^A sites and translate the modification signal into specific biological outcomes. These processes affect many aspects of RNA metabolism, including processing, nuclear export, translation, and stability (Fang et al., 2023; Adamiec and Luciński, 2024).

### 3.1 Writers: methyltransferase complexes

The m^6^A methyltransferase complex catalyses the addition of the m^6^A modification. In animals, this complex comprises several core components: METTL3, METTL14, WTAP, ZC3H13, and VIRMA (Deng and Ma, 2024; Pomaville et al., 2024). Similar proteins have been identified in Arabidopsis: MTA (similar to METTL3), MTB (similar to METTL14), FIP37 (similar to WTAP), VIR (similar to VIRMA), and HAKAI (an E3 ubiquitin ligase). These proteins form the plant m^6^A “writer” complex responsible for m^6^A modification (Jiang et al., 2023; Zhang J. et al., 2024; Ganguly et al., 2025). Studies have shown that these components are required for normal plant development. Loss of *MTA* function arrests embryo development at the globular stage and causes embryo death (Zhong et al., 2008). Knocking out *FIP37* also causes embryo death, whereas partial loss of *FIP37* function results in excessive shoot apical meristem growth (Shen et al., 2016). Plants with mutations in *VIR* and *HAKAI* exhibit defective root and shoot growth and abnormal cotyledons (Růžička et al., 2017). Together, these results demonstrate the critical role of m^6^A modification in many essential processes of plant growth and development.

The expression of these genes is also regulated by abiotic stress. In Arabidopsis, *MTA* expression increases slightly in response to cold, whereas *FIP37* expression increases in response to heat. In rice, *OsFIP* expression increases under cold stress, while *OsMTA*, *OsMTB*, and *OsVIR* decrease under cold, drought or salt stress (Zhang et al., 2019). In tomato, m^6^A writer genes exhibit distinct expression patterns in response to drought, salt, heat, and cold stress (Han et al., 2026). Furthermore, recent studies have identified FIONA1 as a new m^6^A methyltransferase in Arabidopsis and shown that it regulates the transition to flowering by affecting the alternative splicing of *FLC* and the stability of *SPL3* and *SEP3* (Cai et al., 2023). *FIONA1* also participates in responses to salt stress (Cai J. et al., 2024). In apple plants, *MdMTA* helps the plant tolerate drought; this effect is associated with increased mRNA stability and enhanced translation of mRNAs encoding proteins involved in lignin deposition and oxidative stress (Hou et al., 2022). In poplar, *PtrMTA* improves drought tolerance by modulating trichome and root growth. By contrast, *PbrMTA1* in pear acts through changes in chlorophyll content and reactive oxygen species levels (Han C. et al., 2023). The effects of m^6^A writers on downstream gene expression are not consistent across species or stress conditions, but rather depend on the specific transcript, the modification site, and the cellular context. Taken together, these observations suggest that m^6^A writers play a role in plant responses to abiotic stress. However, the causal relationships and functional conservation across species still need to be validated.

### 3.2 Erasers: demethylases

The reversibility of m^6^A modification is achieved through the action of demethylases. In animals, FTO and ALKBH5 are the primary m^6^A demethylases (Liao et al., 2024; Yang et al., 2024). Plant genomes encode multiple ALKBH family proteins, among which ALKBH9B and ALKBH10B have been confirmed to possess m^6^A demethylase activity (Shen et al., 2023; Wang J. et al., 2024; Zhao J. et al., 2024). ALKBH10B has been identified as a key m^6^A demethylase in Arabidopsis, where it plays a role in floral transition by influencing the stability of transcripts such as *SPL3*, *SPL9*, and *FT* (Duan et al., 2017). During alfalfa mosaic virus infection, ALKBH9B modulates m^6^A levels on viral RNA, which in turn affects the systemic infectivity of the virus (Martínez-Pérez et al., 2022).

In recent years, the role of erasers in abiotic stress responses has become increasingly clear. In Arabidopsis, *ALKBH8B* responds to salt and abscisic acid (ABA) (Huong et al., 2022), while *ALKBH9C* is involved in abiotic stress and ABA signalling (Amara et al., 2022). ALKBH9B alters ABA signalling to cope with stress (Tang et al., 2022), whereas ALKBH10B enhances drought tolerance by removing m^6^A and thereby affecting mRNA stability (Han R. et al., 2023). In cotton, GhALKBH10B increases drought tolerance by regulating the expression of genes involved in ABA and calcium signalling (Li B. et al., 2023). In tomato, *SlALKBH10B* negatively regulates seedling tolerance to drought and salt stress (Shen et al., 2023), while SlALKBH2 promotes fruit ripening by altering the stability of *SlDML2* (Zhou et al., 2019). Shi and colleagues concluded that ALKBH6, a potential RNA demethylase, plays a pivotal role in Arabidopsis germination, growth, and survival, and that its function is closely associated with stress responses (Shi et al., 2022). These results indicate that erasers participate in the regulation of plant development and stress responses; however, their effects vary with species, transcript, and stress condition.

### 3.3 Readers: recognition and execution

The biological function of m^6^A modification depends on reader proteins that specifically recognise methylated sites. In animals, YTH domain-containing proteins constitute the primary family of m^6^A readers (Wang and Zhou, 2024; Widmer et al., 2024). In Arabidopsis, ECT2, ECT3, and ECT4 have been identified as functional m^6^A readers (Due Tankmar et al., 2023; Song et al., 2023; Chen et al., 2024). ECT2 regulates 3′ UTR processing in the nucleus; upon export to the cytoplasm, it binds to m^6^A-containing RNAs, promoting their translation or directing them to stress granules under stress conditions (Scutenaire et al., 2018; Wei LH. et al., 2018). ECT3 and ECT4 are involved in regulating the timing of leaf morphogenesis (Arribas-Hernández et al., 2020).

The role of m^6^A readers in abiotic stress responses is becoming increasingly clear. In Arabidopsis, *ECT8* is an m^6^A reader implicated in abiotic stress responses and is proposed to accelerate mRNA decay (Cai Z. et al., 2024). ECT12 affects abiotic stress responses in Arabidopsis by altering mRNA stability (Amara et al., 2024). In tomato, SlYTH1 is a putative m^6^A reader that influences plant growth and fruit shape (Yin et al., 2022). SlYTH2 reduces tomato fruit aroma by blocking translation (Bian et al., 2024). *SlYTP8* and *SlYTP9* are involved in responses to low-temperature and waterlogging stress in tomato (Zhang Y. et al., 2024). Shi et al. observed that, under abiotic stress, the expression of m^6^A readers fluctuates more than that of writers and erasers, suggesting that decoding methylation marks may be more important than adding or removing them during plant stress adaptation (Shi et al., 2022). Reader-mediated recognition of m^6^A marks plays an important role in stress adaptation; however, its effects vary with species, transcript, and stress condition.

### 3.4 The three classes of proteins coordinately constitute a regulatory network

Writers, erasers, and readers do not work alone. Writers add methyl groups to specific sequence motifs, such as the RRACH motif; erasers remove these marks; and readers recognise methylated sites and convert the modification signal into specific biological effects. This network also exhibits spatial specialisation: methylation primarily occurs in the nucleus (Shinde et al., 2023); demethylases are present in both the nucleus and the cytoplasm (Wei J. et al., 2018); and readers perform distinct functions in both locations (Scutenaire et al., 2018; Ao et al., 2023; Cui et al., 2024). These three protein types work together to dynamically control RNA processing, nuclear export, translation, and stability, thereby forming a finely tuned post-transcriptional regulatory network. This network plays a crucial role in plant growth, development, and stress responses (Lin et al., 2023; Tayier et al., 2024; Mudasir et al., 2026).

Research into this network in crops has advanced gradually in recent years. During tomato fruit development, m^6^A modification influences fruit expansion by regulating the expression of genes associated with the cell cycle and auxin signaling. It also affects ripening by regulating the expression of genes associated with transcription factors, ethylene biosynthesis and signaling, and lycopene biosynthesis. Under drought, salt, heat, or cold stress, m^6^A modification regulates the transcript levels of these genes by modulating mRNA stability, thereby affecting fruit expansion and ripening (Han et al., 2026). In apple, MhYTP1 and MhYTP2 act as potential m^6^A readers and promote fruit ripening (Wang et al., 2017). In strawberry, MTA-mediated m^6^A modification promotes ripening by increasing the stability of *NCE05* and *AREB1* mRNAs (Zhou et al., 2021). Shi and colleagues were the first to systematically summarize the role of RNA methylation in stress-regulated flowering. They highlighted that m^6^A modification regulates stress-induced gene expression to balance reactive oxygen species and ATP levels and affects the flowering transition by regulating flowering-related factors such as *SPL* and *FT*. They also demonstrated that erasers such as ALKBH6 mediate RNA methylation and demethylation under stress conditions (Shi et al., 2022).

These studies demonstrate that the m^6^A regulatory network is both conserved and diverse with respect to plant development and stress responses. The core m^6^A regulatory machinery is broadly conserved across plant species. For example, MTA and FIP37 act as core subunits of the methyltransferase complex and are required for normal development and stress responses in Arabidopsis, rice, and apple. At the same time, apparent functional differences exist among species. Take ALKBH10B as an example. In Arabidopsis, it mainly regulates flowering time by modulating the stability of *FT*, *SPL3*, and *SPL9* transcripts (Duan et al., 2017). In cotton and tomato, however, its orthologs are primarily involved in drought or salt stress tolerance (Li B. et al., 2023; Shen et al., 2023), and no obvious flowering defects have been reported in knockout lines, suggesting that this eraser has adopted distinct roles in crops. Similarly, overexpression of *MdMTA* in apple enhances drought tolerance (Hou et al., 2022), whereas loss of *MTA* function in Arabidopsis causes embryonic lethality (Zhong et al., 2008), highlighting how the same factor can produce drastically different phenotypes across species. At the reader level, Arabidopsis ECT2, ECT3, and ECT4 mainly stabilize target mRNAs to regulate leaf and trichome development (Wei LH. et al., 2018; Arribas-Hernández et al., 2020; Song et al., 2023), whereas tomato SlYTH2 negatively affects fruit aroma volatiles by repressing translation (Bian et al., 2024). This suggests that reader functions also differ among plant species. Overall, while the biochemical activities of writers, erasers, and readers are relatively conserved, their downstream targets, tissue-specific expression, and phenotypic consequences have diverged considerably. This divergence likely reflects changes in downstream regulatory networks and target gene repertoires during evolution, rather than differences in the intrinsic biochemical properties of the proteins themselves. This is an important caveat when translating knowledge from model plants to crop improvement.

Collectively, these proteins establish the fundamental principles of the m^6^A regulatory network. With this framework, it is possible to explore in greater depth how writers, erasers, and readers coordinate to control stress responses at the RNA level. In essence, m^6^A controls stress-related gene expression through multiple mechanisms, including mRNA stability, translational regulation, splicing, and polyadenylation. This process depends on interactions among writers, erasers, and readers in different contexts (**Figure 2**).

**Figure 2:**
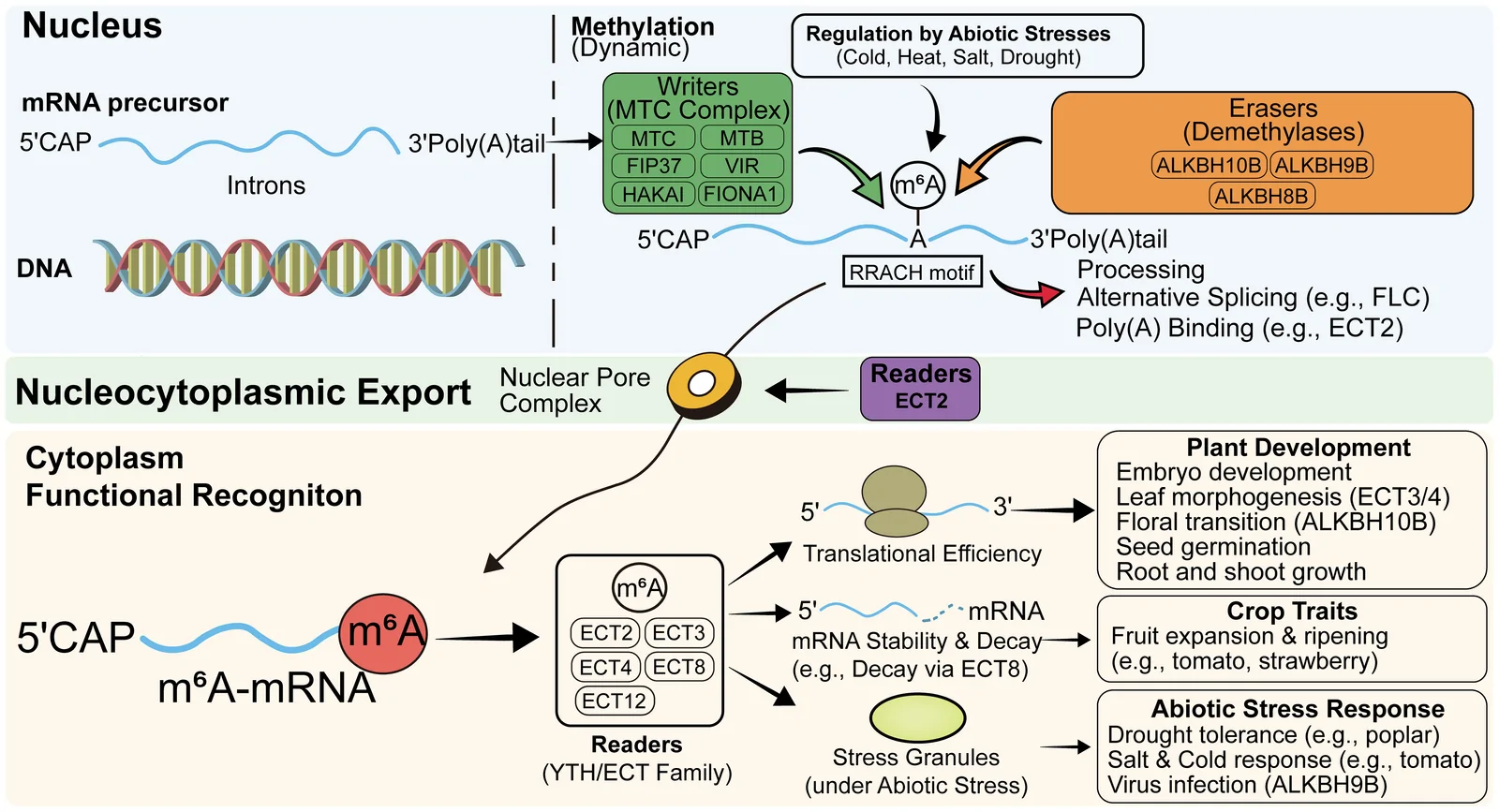
Composition and coordination of the plant m^6^A regulatory network. m6A modification relies on methyltransferase complexes (e.g., MTA, MTB, FIP37) and demethylases (e.g., ALKBH9B/10B) in the nucleus, maintaining a dynamic balance subject to stress signals. After nuclear export, modified mRNAs are recognized by YTH/ECT family readers. These factors participate in mRNA processing, export, translation, and stability regulation, which collectively affect plant development, crop traits, and stress responses.

## 4 Molecular mechanisms of m^6^A-mediated abiotic stress responses in plants

### 4.1 m^6^A regulates stress-responsive gene expression by modulating mRNA stability

Accumulating evidence indicates that controlling mRNA stability is a major mechanism by which m^6^A influences stress responses, although other regulatory layers may also contribute. For instance, under high salinity or drought, m^6^A writers and erasers respond by adding or removing these marks, and readers then interpret them to determine the decay rate of specific stress-responsive transcripts. This enables rapid, precise changes in gene expression.

The writer complex plays a key role here, and its effects depend on context. In Arabidopsis, the methyltransferase VIR mainly adds m^6^A to the 3′ UTR of target mRNAs. When *VIR* is lost, m^6^A levels decrease, and the stability of several negative salt tolerance regulators—including *ATAF1*, *GSTU17*, and *GI*—increases significantly, which is associated with a salt-hypersensitive phenotype (Hu et al., 2021). Another methyltransferase, FIONA1 (FIO1), operates differently. In *fio1* mutants, the stability of positive salt stress regulators such as *AOX1a* decreases, whereas the stability of the negative regulator *GSTU17* increases; together, these changes result in a salt-sensitive phenotype (Cai J. et al., 2024). These observations suggest that different writers may exert opposite effects on the same target gene, possibly through methylation at distinct sites on the transcript, although direct evidence is still needed. Beyond these writer-specific functions, the core writer component MTA has also been extensively studied in relation to stability control. In Arabidopsis, m^6^A stabilizes mRNA by preventing local cleavage by ribonucleases, thereby providing direct protection against endonucleolytic attack (Anderson et al., 2018). This protective role has been confirmed in stress responses of other plant species. For instance, *MdMTA* overexpression in apple increases the stability of drought-responsive transcripts and improves drought tolerance (Hou et al., 2022). In strawberry, *MTA* overexpression also increases mRNA stability during fruit ripening, although its role in stress responses remains unclear (Zhou et al., 2021).

Studies across species show that writers and erasers may have either conserved or distinct functions. In poplar, for example, the methyltransferase *PagFIP37* is induced by salt and stabilizes positive regulators such as *PagMYB48* and *PagGT2* to promote salt tolerance (Zhao Y. et al., 2024). Conversely, research on the rice demethylase OsALKBH5 reveals a different aspect: OsALKBH5 is required for normal male meiosis. When *OsALKBH5* is lost, several meiotic genes, including *OsCOM1* and *OsZIP4*, become hypermethylated and less stable, leading to defective double-strand break repair and possible male sterility (Xue et al., 2024). In Arabidopsis, the demethylase ALKBH10B is involved in salt stress and ABA responses by altering the stability of target mRNAs (Shoaib et al., 2021). Reader proteins recognize the m^6^A mark and directly influence the fate of modified transcripts. In Arabidopsis, the ECT2 reader controls trichome shape by regulating target mRNA stability (Wei LH. et al., 2018), while ECT8 functions similarly in salt stress and ABA responses (Cai Z. et al., 2024). Recently, ECT10 was found to bind directly to m^6^A-modified drought-responsive transcripts. In *ect10* mutants, the stability of positive drought regulators such as *RD21A* and *DI21* increases, whereas that of negative regulators such as *PUB11* and *SMXL6* decreases, ultimately improving drought tolerance (Han et al., 2025). Interestingly, ECT12 has the opposite effect under drought stress (Amara et al., 2024). It has been proposed that different ECT family readers may recruit distinct interacting proteins and thereby exert opposing effects on target mRNA stability, although this model requires further testing.

The effect of m^6^A on mRNA stability varies by transcript and depends on several factors. One factor is the location of the modification. In poplar, for example, hypermethylation in the coding sequence (CDS) tends to be associated with higher transcript levels, but this is not necessarily the case for m^6^A in the 3′ UTR (Gao et al., 2021). The outcome also depends on the context and the reader protein involved. In mammals, YTHDF2 and YTHDF3 promote mRNA degradation by recruiting the CCR4-NOT deadenylase complex, whereas IGF2BP proteins increase mRNA stability (Micaelli et al., 2022; Sun et al., 2024). Whether similar opposing mechanisms operate in plants remains unclear. In sugar beet, approximately 80% of genes with changes in both methylation and expression show a negative trend, characterized by higher m^6^A levels and lower transcript abundance (Li J. et al., 2023). This contrasts with the positive correlation observed for certain genes in Arabidopsis and poplar. Therefore, m^6^A regulatory mechanisms are diverse across plants. In addition, m^6^A can interact with other regulatory processes. In Arabidopsis, for example, m^6^A added under salt stress is linked to reduced mRNA secondary structure; this structural change may facilitate binding of specific readers or directly affect nuclease access to RNA (Kramer et al., 2020).

In plants, m^6^A therefore controls mRNA stability, which serves as a major route for regulating stress-responsive gene expression. Methylation (writers), demethylation (erasers), and recognition (readers) all contribute, each adding a distinct layer of control. Whether a transcript becomes more or less stable depends on the context—including the gene, the position of the m^6^A mark, the proteins involved, and the plant species. Overall, some conserved rules emerge alongside differences among plant families. It should be noted that transcriptome-wide correlations between m^6^A abundance and transcript levels do not by themselves establish altered mRNA stability; direct measurements of RNA half-life or targeted manipulation of m^6^A writers/erasers provide stronger evidence. Of the studies cited in this section, those employing RNA half-life measurements or genetic manipulation of m^6^A writers/erasers were considered to provide mechanistic validation, whereas those based solely on m^6^A-seq correlations were treated as associative evidence.

### 4.2 m^6^A-mediated translational regulation under stress conditions

In addition to its effect on mRNA stability, m^6^A promotes stress adaptation by adjusting translation efficiency under conditions such as drought, cold, and salt stress. The relationship between translation efficiency and m^6^A modification has been demonstrated in various plant species. In maize, for example, polysome profiling has revealed that the effect of m^6^A on translation depends not only on its presence but also on its location and abundance. When m^6^A levels are low near the start codon or in the 3′ UTR, translation tends to increase; however, when m^6^A is too abundant in the 3′ UTR, translation decreases (Luo et al., 2020). In strawberry fruit, the methyltransferase FvMTA increases both mRNA stability and the translation efficiency of key ABA pathway genes, thereby helping to control fruit ripening (Zhou et al., 2021). In apple leaves, the m^6^A reader MhYTP2 recognizes m^6^A marks, altering target mRNA stability and increasing the translation efficiency of antioxidant genes, which provides resistance to powdery mildew (Guo et al., 2022).

The role of m^6^A in translational control under cold stress is well understood. In Arabidopsis, for example, cold treatment reduces global m^6^A levels, primarily in the 3′ UTR. However, transcripts with cold-enriched m^6^A peaks exhibit higher translation efficiency at low temperatures, suggesting that m^6^A facilitates the translation of certain transcripts to mediate cold adaptation (Govindan et al., 2022). Genetic evidence supports this: the MTA-deficient mutant (*mta*) is more sensitive to cold, and translation efficiency changes for approximately one-third of the genome. One cold-responsive gene, *DGAT1*, exhibits reduced translation efficiency in *mta* mutants, and loss of *DGAT1* also causes cold sensitivity, indicating that m^6^A-mediated translational control is necessary for cold tolerance (Wang et al., 2023). The role of m^6^A in translational control under drought stress is also becoming clearer. In Arabidopsis, drought stress causes global m^6^A remodeling, and MTA-dependent m^6^A changes are closely linked to the control of translation efficiency. Loss of *MTA* leads to incorrect translation efficiency for approximately 7,000 genes under drought conditions, many of which are involved in ribosome biogenesis, mRNA processing, or protein translation. There is also some evidence that MTA might affect translation independently of m^6^A, for example by altering RNA polymerase II transcription elongation (Ganguly et al., 2025). In cotton, the m^6^A demethylase GhALKBH10B reduces drought tolerance by removing m^6^A from transcripts in the ABA and Ca²⁺ signaling pathways, accelerating their decay (Zhang D. et al., 2024). Thus, the relationship between m^6^A levels and translation efficiency appears to be complex.

At the molecular level, m^6^A readers play a key role in directly influencing translation initiation and elongation. In mammals, YTHDF1 binds eIF3 to promote translation initiation, whereas YTHDF2 and YTHDF3 recruit deadenylase complexes to balance translation with degradation (Zhang S. et al., 2025; Zhao et al., 2025). In plants, the m^6^A readers ECT2, ECT3, and ECT4 stabilize target mRNAs by directly recruiting poly(A)-binding proteins and are involved in ABA signaling and drought responses (Song et al., 2023). Additionally, ECT8 forms phase-separated condensates under ABA and drought stress, thereby controlling ABA signaling pathways (Wu et al., 2024). These findings demonstrate that plant m^6^A readers employ various mechanisms to regulate translation. Studies in wheat reveal even greater complexity: m^6^A-containing transcripts are highly enriched in pathways associated with “translation” and “RNA transport,” encompassing ribosomal proteins, translation initiation factors, and nuclear export factors (Huang et al., 2022). The authors suggested that m^6^A may exert a second level of control over translation by directly altering the translation efficiency of genes encoding translation machinery components, enabling rapid responses to environmental changes. This view is supported by research on rice, in which both biotic (e.g., viral infection) and abiotic (e.g., cadmium stress) stresses cause significant changes in the m^6^A modification of translation-related genes (Wang P. et al., 2021; Zhang et al., 2021). Position-dependent effects of m^6^A on translation efficiency have also been observed in ornamental plants. In roses, for example, light treatment reduced expression of the demethylase genes *RhALKBH10A* and *RhALKBH10B*, resulting in increased m^6^A levels in the CDS region of the *RhANS* transcript and decreased levels in the 3′ UTR. CDS modification increased *RhANS* translation efficiency, whereas 3′ UTR modification had the opposite effect, demonstrating that the impact of m^6^A on translation depends on its location within the transcript (Gao et al., 2026). In rice, overexpression of the moso bamboo methyltransferase genes *PheMTA1* and *PheMTA2* not only promoted root growth but also increased salt tolerance; these methyltransferases bind to transcripts involved in stress responses (Wang et al., 2025).

In summary, studies in various plant species have shown that m^6^A modification modulates both mRNA stability and translation efficiency under stress conditions. Several patterns emerge from these observations. First, the effect of m^6^A on both stability and translation depends on its position within the transcript, with methylation in the CDS and 3′ UTR often associated with distinct regulatory outcomes. Second, reader proteins are consistently involved in translating m^6^A signals into biological outputs, whether by recruiting poly(A)-binding proteins to stabilize mRNAs or by forming phase-separated condensates. Third, m^6^A can coordinate mRNA stability and translation efficiency to achieve rapid regulation of stress-responsive gene expression. These patterns provide a useful framework for understanding m^6^A functions in plant stress adaptation and a reference for targeted manipulation in crop breeding. Beyond mRNA stability and translation, m^6^A has also been reported to participate in stress responses through additional mechanisms, as discussed in the following section.

### 4.3 m^6^A participates in stress responses through multiple mechanisms

In addition to influencing mRNA stability and translation, m^6^A affects stress responses through other mechanisms, including alternative splicing, alternative polyadenylation, and the movement of mRNA into stress granules. Alternative splicing is an important mechanism by which plants respond to abiotic stress. Cai and colleagues demonstrated that FIONA1, an m^6^A methyltransferase in Arabidopsis, controls the timing of flowering by altering the alternative splicing of *FLC*, a major flowering repressor, thereby changing the ratio of different *FLC* transcript variants. Because *FLC* integrates temperature signals such as vernalization, this regulatory mechanism may be associated with temperature stress responses (Cai et al., 2023). In a study of heat stress in rice, Qadir and colleagues examined m^6^A regulatory genes in three rice species: *Oryza sativa* subsp. japonica, *Oryza sativa* subsp. indica, and *Oryza glaberrima*. They found that the expression of these genes changes under heat stress. The authors also examined promoter regions for cis-regulatory elements and identified several stress-related motifs—ABRE, G-box, and TCA—all of which are tightly linked to ABA signaling and stress responses (Qadir et al., 2025). This suggests that m^6^A regulators themselves may respond to stress signals, possibly through changes in expression or alternative splicing. Consider a study of salt stress in maize. He and colleagues used ONT DRS to profile changes in root transcripts under salt stress. Some alternative splicing events (A3, RI, A5, and SE) became less frequent. APA site usage also shifted: fewer APA sites per transcript were associated with lower abundance, whereas more sites were associated with higher abundance. The number of m^6^A modification sites also decreased (He et al., 2024). Taken together, these observations suggest that salt stress could influence alternative splicing and polyadenylation by altering m^6^A patterns.

Stress granules are atypical organelles that lack a membrane. In plant cells, they form in response to adverse environmental conditions, temporarily store mRNAs, and delay translation. For example, in a study on hypoxic stress, Kubiak and colleagues closely observed the formation of stress granules in lupin roots. They also found that in Arabidopsis, under low oxygen levels, the m^6^A reader protein ECT2 moves to stress granules (Kubiak et al., 2025). This study built on earlier work showing that YTH domain-containing proteins, which recognize m^6^A, are found in stress granules in Arabidopsis (Kosmacz et al., 2019; Tao et al., 2025). In lupin, the researchers found that m^6^A modification increased during the early stages of stress granule assembly but decreased as stress continued. Using an Arabidopsis mutant with reduced m^6^A levels (pABI3::MTA-*mta*), they demonstrated that lower m^6^A levels result in fewer stress granules, less poly(A) RNA within them, and reduced ECT2 accumulation in stress granules. Thus, m^6^A modification appears to promote stress granule assembly and influence mRNA movement within the cell, both of which are integral to plant stress responses. For example, overexpression of the moso bamboo methyltransferase genes *PheMTA1* and *PheMTA2* in rice altered the alternative splicing of stress-related genes such as *OsRS33*, *OsPRR73*, and *OsHAP2E*, thereby affecting stress responses (Wang et al., 2025). Similarly, in ginkgo, treatments with ABA, methyl jasmonate, and NaCl markedly altered the expression of *GbYTH* genes, suggesting that YTH family reader proteins may be involved in abiotic stress responses (Wang H. et al., 2024). In Masson pine, 22 m^6^A regulatory genes were identified—seven writers, seven erasers, and eight readers—and their expression changed significantly under ABA treatment and drought stress, suggesting that m^6^A regulatory genes may be involved in stress responses (Yao et al., 2024). Overall, control of mRNA stability and translation is not the only mechanism involved; m^6^A also contributes to abiotic stress responses through alternative splicing, alternative polyadenylation, and mRNA trafficking to stress granules. It should be noted that in many of these studies, the observed correlations between m^6^A changes and splicing or polyadenylation events do not establish causality; direct manipulation of m^6^A at specific sites is needed to confirm its role. For stress granule studies, evidence for m^6^A involvement ranges from localization or association to functional effects; studies that directly demonstrate m^6^A-regulated stress granule formation (e.g., changes in stress granule number or composition upon m^6^A level alteration) provide stronger evidence than those reporting only co-localization of m^6^A modification with stress granules. Similarly, expression-based observations should be distinguished from studies that include genetic or biochemical validation. Understanding these mechanisms, while recognizing the current limitations in causal evidence, provides a foundation for exploring the use of m^6^A regulatory elements in crop stress tolerance (**Figure 3**).

**Figure 3:**
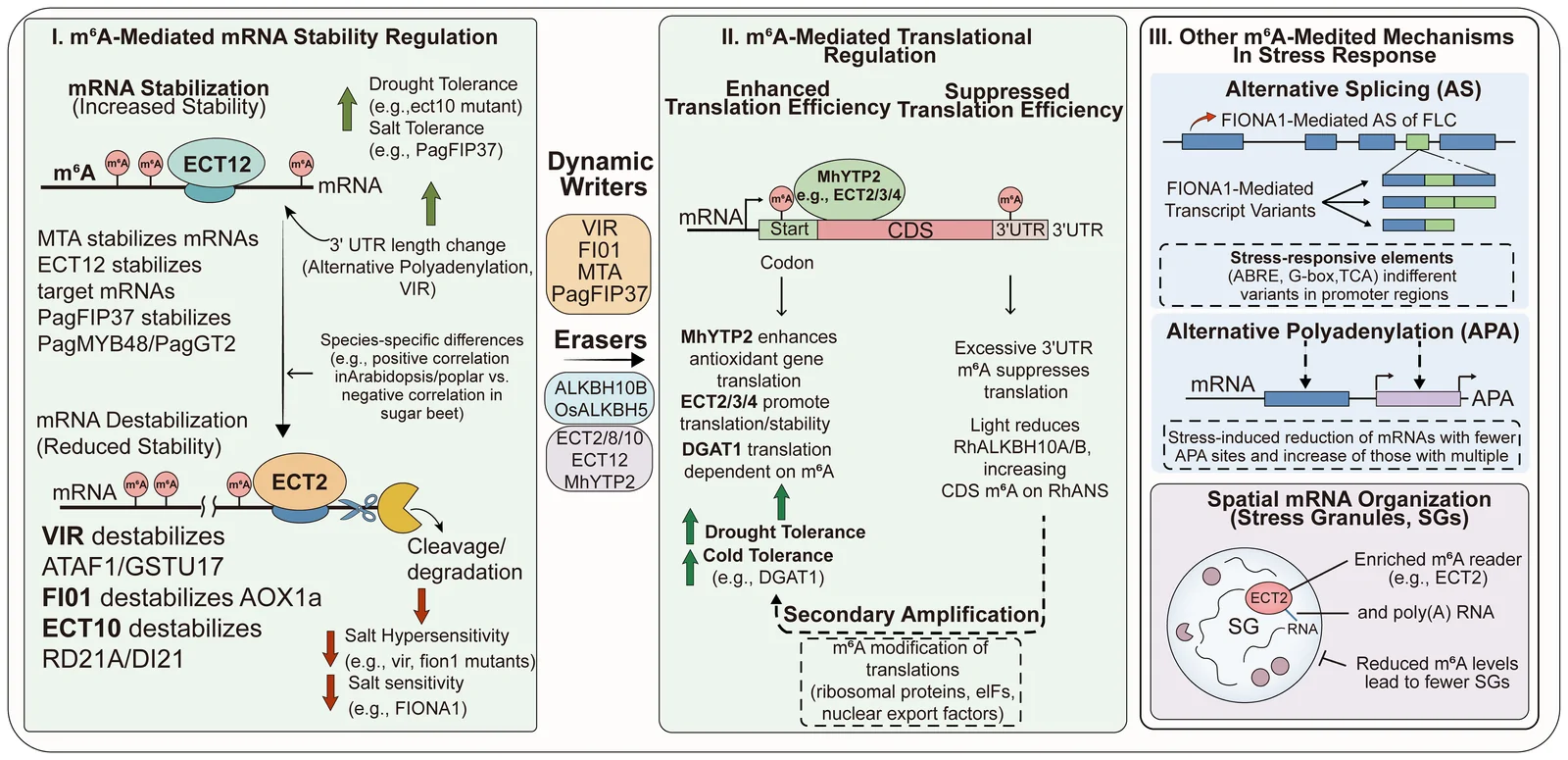
m^6^A participates in plant stress responses through regulating mRNA stability, translation efficiency, and multiple mechanisms. Three major pathways are summarized. (I) mRNA stability regulation: Writers and erasers are involved in modulating the stability of target transcripts, potentially associated with species-specific tolerance phenotypes. (II) Translational regulation: Certain readers (e.g., MhYTP2, ECT2/3/4) contribute to the translation efficiency of target genes under drought or cold stress. (III) Other mechanisms: These include m^6^A-participated alternative splicing, alternative polyadenylation (APA), and mRNA sorting into stress granules. Solid arrows indicate experimentally established interactions; dashed arrows indicate inferred or proposed relationships; dashed boxes indicate inferred mechanisms or components (based primarily on transcriptome associations or bioinformatics predictions) that require further validation.

## 5 Application potential and strategies of m^6^A in crop stress-resistance breeding

Given the central role of m^6^A in stress-responsive mechanisms, recent studies have identified multiple m^6^A regulatory factors across various crops; these factors show promise for breeding applications because they have significant effects on stress tolerance and cause minimal developmental side effects.

### 5.1 m^6^A regulators with application potential in crop stress-resistance breeding

Recent studies, spanning model plants to various crops, have identified multiple m^6^A regulatory factors involved in stress responses, several of which show promise for breeding applications. In apple, overexpression of the m^6^A methyltransferase *MdMTA* enhances drought tolerance without obvious growth defects, whereas *MdMTA*-RNAi plants show developmental retardation (Hou et al., 2022). Thus, *MdMTA* appears to have potential value for drought-resistance breeding. In pear, silencing *PbrMTA1* significantly reduces drought tolerance, further confirming the conserved function of *MTA* family members in woody fruit trees (Han C. et al., 2023). In switchgrass, characterization of the m^6^A gene family revealed that *PvALKBH10* is involved in flowering regulation, suggesting a potential role in stress adaptation (Liu H. et al., 2024). In cotton, silencing the m^6^A demethylase *GhALKBH10B* significantly enhances drought tolerance without apparent growth inhibition under normal conditions, identifying this gene as a potential target for negative regulation (Li B. et al., 2023). Similarly, knocking out *SlALKBH10B* in tomato enhances both drought and salt stress tolerance, with mutants growing normally under control conditions (Shen et al., 2023). In tobacco, overexpressing the m^6^A methyltransferase *NtFIP37B* improves drought resistance without obvious growth defects (Su et al., 2024). Expressing the human m^6^A demethylase *FTO* in rice greatly increases yield and drought tolerance, promotes root growth and photosynthetic efficiency, and causes no adverse side effects. This finding provides the first demonstration that manipulating m^6^A levels can improve crops and represents a breakthrough (Yu et al., 2021). In soybean, overexpression of *GmMTA2* and *GmMTB1* helps plants tolerate alkalinity and darkness. Furthermore, m^6^A sites in soybean roots under cadmium stress have been identified, providing potential targets for breeding (Zhang et al., 2023; Liu P. et al., 2024; Ma et al., 2024). In wheat, the m^6^A reader *TaETC9* contributes to drought tolerance, and loss of this gene substantially reduces drought resistance (Pan et al., 2024). In sweet sorghum, salt stress-induced m^6^A modification is closely associated with salt tolerance. Salt-sensitive varieties show enhanced m^6^A modification on key salt-responsive transcripts, such as *SbIAR4* and *SbNRT1.5*, under salt stress, and this increase correlates with increased mRNA stability (Zheng et al., 2021). In watermelon, overexpression of the m^6^A methyltransferase *ClMTB* enhances drought tolerance, an effect that may be attributed to alleviation of oxidative stress and maintenance of photosynthetic capacity (He et al., 2021). In poplar, *PagFIP37* positively regulates salt tolerance by enhancing the stability of salt-responsive transcripts, including *PagMYB48*, *PagGT2*, and *PagNAC2* (Zhao Y. et al., 2024). In sugar beet, salt stress induces substantial changes in the m^6^A landscape, and hypomethylation correlates with increased expression of key salt-tolerance genes and enhanced mRNA stability (Li J. et al., 2023).

Together, these findings suggest several feasible breeding strategies, including overexpression of methyltransferases such as *MTA* and *FIP37*, knockout of demethylases such as *ALKBH10B*, and heterologous expression of *FTO*. In several cases, stress tolerance was enhanced without obvious growth defects under controlled conditions, suggesting that these regulators have potential value in breeding. However, the absence of reported side effects in specific studies should not be generalized, because m^6^A regulators are broadly involved in development and can cause pleiotropic phenotypes depending on the gene, species, and manipulation strategy. These strategies can be grouped into three main categories. It should be noted that most of these examples represent proof-of-concept studies (e.g., heterologous *FTO* expression in rice) rather than validated breeding applications. Similarly, the sugar beet hypomethylation entry in **Table 1** is based on correlative evidence rather than gene-specific manipulation and thus is not directly comparable with the other entries.

**Table 1:** Breeding potential of major m^6^A regulators across different crop species.

Species	Regulator	Type	Stress response	Evidence and validation	Breeding strategy
Apple	MdMTA (Hou et al., 2022)	Writer	Drought	Overexpression enhances drought tolerance; silencing reduces tolerance (Functional validation)	Overexpression
Pear	PbrMTA1 (Han C. et al., 2023)	Writer	Drought	Silencing reduces drought tolerance (Functional validation)	Overexpression
Poplar	PagFIP37 (Zhao Y. et al., 2024)	Writer	Salt	Overexpression enhances salt tolerance (Functional validation)	Overexpression
Cotton	GhALKBH10B (Li B. et al., 2023)	Eraser	Drought	Silencing enhances drought tolerance (Functional validation)	Silencing/Knocout
Tomato	SlALKBH10B (Shen et al., 2023)	Eraser	Drought /Salt	Knockout enhances drought and salt tolerance (Functional validation)	Knockout
Tobacco	NtFIP37B (Su et al., 2024)	Writer	Drought	Overexpression enhances drought resistance (Functional validation)	Overexpression
Rice	FTO (Yu et al., 2021)	Eraser	Drought	Heterologous expression increases yield and drought tolerance in field trials (Functional validation, field)	Heterologous expression
Soybean	GmMTA2/GmMTB1 (Zhang et al., 2023; Liu P. et al., 2024; Ma et al., 2024)	Writer	Alkalinity/Dark	Overexpression improves tolerance to alkalinity and darkness (Functional validation)	Overexpression
Wheat	TaETC9 (Pan et al., 2024)	Reader	Drought	Knockout reduces drought resistance (Functional validation)	Overexpression
Sorghum	SbMTA (Zheng et al., 2021)	Writer	Salt	Upregulation correlates with enhanced m^6^A modification and salt resistance (Genetic correlation)	Overexpression
Watermelon	ClMTB (He et al., 2021)	Writer	Drought	Overexpression in tobacco enhances drought tolerance (Heterologous validation)	Overexpression
Sugar beet	hypomethylation (Li J. et al., 2023)	—	Salt	Hypomethylation correlates with increased salt-tolerance gene expression and mRNA stability (Genetic correlation)	Breeding selection

“Functional validation” refers to direct evidence from transgenic or mutant analyses in the same species; “heterologous validation” refers to evidence from overexpression in a different species; and “genetic correlation” refers to evidence based on correlative changes in expression or methylation without direct functional validation. The verbs used in the evidence descriptions themselves indicate the direction of regulation; for example, “enhances” indicates positive regulation, whereas “reduces” indicates negative regulation.

### 5.2 Genetic improvement strategies for crop stress resistance based on m^6^A regulation

Broadly speaking, strategies that use m^6^A regulation to enhance crop stress resistance can be categorized into three main groups. One approach is precision editing. Recent advances in CRISPR/dCas13 systems fused with m^6^A methyltransferases or demethylases have made it possible to adjust m^6^A modification on individual transcripts with great precision. In Arabidopsis, for example, an m^6^A editing system combining dCas13a with MTA achieved precise regulation of m^6^A levels on the *SHR* transcript, which in turn affected root development (Shi et al., 2024). In cotton, a targeted RNA methylation editor (CRISPR/dCas13(Rx) fused with GhMTA) successfully increased m^6^A levels on the *GhDil9* transcript, resulting in improved root development and drought resistance (Yu et al., 2024). In rice, CRISPR-based editing systems targeting m^6^A regulatory genes such as *OsALKBH5* have provided insights into the influence of m^6^A on meiotic progression and male fertility (Xue et al., 2024). The advantage of such strategies lies in their ability to precisely target key stress-responsive genes, avoiding the developmental side effects associated with global changes in m^6^A levels.

However, CRISPR-based m^6^A editing systems still have technical limitations. Off-target activity is a major concern. When dCas13 is used for RNA targeting, the guide RNA can bind to transcripts that are not fully complementary, which may result in unwanted m^6^A modifications at unintended sites (Abudayyeh et al., 2016; Abudayyeh et al., 2017). Recent work in plants has evaluated the specificity of dCas13-based m^6^A editors through transcriptome-wide m^6^A profiling (Fang et al., 2023; Shi et al., 2024; Yu et al., 2024). These studies found only limited off-target effects in the edited lines, suggesting that the dCas13 system can achieve reasonable specificity under the conditions tested. Nevertheless, the off-target profile of dCas13-based m^6^A editors has not yet been systematically examined across different species or target transcripts; this remains an open question for future investigation. In addition, the catalytic domains attached to dCas13 may themselves contribute to off-target activity, independent of the binding specificity of dCas13 itself (Li et al., 2020; Wilson et al., 2020). Possible ways to reduce off-target effects include refining guide RNA design, using higher-fidelity dCas13 variants, and delivering the editors as ribonucleoprotein complexes. These strategies have shown promise in other CRISPR systems and deserve further testing in the context of plant m^6^A editing.

Another strategy involves broader genetic manipulation, such as overexpressing beneficial m^6^A regulators or knocking out negative ones. Numerous examples illustrate this approach. In apple, overexpression of *MdMTA* enhances drought tolerance (Hou et al., 2022). In cotton, silencing *GhALKBH10B* enhances drought tolerance (Li B. et al., 2023). Tomato plants lacking *SlALKBH10B* exhibit improved tolerance to both drought and salt stress (Shen et al., 2023). Tobacco overexpressing *NtFIP37B* has improved drought resistance (Su et al., 2024). Conversely, silencing *PbrMTA1* in pear reduces drought tolerance. Poplar overexpressing *PagFIP37* has enhanced salt tolerance (Zhao Y. et al., 2024). In rice, heterologous expression of *FTO* significantly increases yield and biomass in field trials (Yu et al., 2021). In sorghum, increasing *SbMTA* boosts m^6^A modification and salt resistance (Zheng et al., 2021), whereas overexpressing *SbALKBH10B* has the opposite effect. These strategies have the advantage of being relatively simple to implement and make it possible to screen transgenic lines with both enhanced stress tolerance and normal development.

### 5.3 Current challenges and future research directions

The potential of m^6^A regulation for breeding stress-tolerant crops is clear. However, challenges remain. A key challenge is achieving stress-specific m^6^A regulation so that developmental abnormalities can be avoided. m^6^A modification is widely involved in plant growth and development, and global changes in m^6^A levels often lead to pleiotropic phenotypes. For example, loss of *MTA* function causes abnormal embryo development in Arabidopsis (Zhong et al., 2008). Similarly, disrupting *OsFIP37* in rice causes abnormal pollen growth and male sterility (Zhang et al., 2019). Therefore, future work should aim to build m^6^A regulatory systems that are activated by stress-inducible promoters or that can be controlled only in certain tissues, such as roots or leaves, at specific times.

Beyond technical performance, regulatory and public considerations also warrant attention. Crops developed through epigenome editing may be subject to regulatory frameworks that are still taking shape. One example is the European Union, where a 2018 Court of Justice ruling placed most genome-edited plants under existing GMO legislation—a move that could hinder their transition from the laboratory to the field. At the same time, national approaches are not uniform; the United States and Argentina, for instance, have each formulated separate policies for edited products that contain no foreign DNA (Dalakouras and Vlachostergios, 2021). Beyond regulatory classification, the economic feasibility of m^6^A-based breeding depends on multiple factors, including the cost of CRISPR/dCas13 system development, plant transformation efficiency, and scale of application. Current CRISPR-based editing systems require substantial upfront investment in guide RNA design, construct optimization, and transformation pipeline establishment. However, once established, these systems can be applied across multiple target genes and crop species, potentially reducing per-trait costs. Compared with conventional breeding, which typically requires multiple generations of crossing and selection over several years, m^6^A editing could theoretically accelerate trait improvement through targeted modulation of regulatory mechanisms without altering the underlying DNA sequence. Nevertheless, a comprehensive cost-benefit analysis must account for species-specific transformation efficiencies, which remain low in many recalcitrant crops, as well as regulatory compliance costs that vary by jurisdiction.

Public perception also represents an important consideration. Unlike transgenesis, which introduces foreign DNA into the genome, m^6^A editing via CRISPR/dCas13 operates at the RNA level and does not create permanent changes to DNA sequences. This distinction is important when considering regulatory frameworks that differentiate between DNA-level and RNA-level modifications. However, the absence of permanent DNA changes does not guarantee heritable breeding effects, particularly in seed-propagated crops, where RNA-level modifications are not necessarily transmitted to the next generation. For vegetatively propagated crops, this limitation may be less relevant. The novelty of epitranscriptomic editing may raise unfamiliar questions about safety and heritability. Experience from the introduction of genetically modified crops suggests that transparent communication, rigorous safety assessment, and clear labeling policies are essential for building public trust (Johannes et al., 2009). Engaging stakeholders—including farmers, consumers, and policymakers—early in the development process may facilitate broader acceptance of m^6^A-based breeding products.

Another issue of practical relevance for breeding is whether induced epigenetic changes can be stably inherited over generations. In plants, CG and CHG methylation can be sustained via MET1 and CMT3 activity, whereas CHH methylation relies largely on *de novo* RdDM and often fades in successive generations unless continuously triggered (Johannes et al., 2009). Certain DNA methylation alterations do persist across multiple generations, as observed in Arabidopsis epiRILs and soybean lines derived from MSH1 (Reinders et al., 2009; Raju et al., 2018; Tonosaki et al., 2022). By contrast, m^6^A modification acts at the RNA level, so its transgenerational behavior likely differs from that of DNA methylation. Currently, knowledge in this area remains limited, and the stability of m^6^A marks probably varies considerably depending on the target locus, type of stress, and environmental conditions. For vegetatively propagated crops, the hurdle of meiotic transmission can be avoided (Dawane et al., 2024); however, for seed-propagated crops, securing reliable inheritance of desirable epialleles remains a major obstacle.

A second challenge is that crops in nature often face multiple stresses simultaneously. We still do not understand how m^6^A functions under these combined stress conditions. In rice, m^6^A modification patterns differ under drought and salt stress (Wang et al., 2022), and the co-occurrence of virus infection and abiotic stress may involve complex m^6^A regulatory networks. In maize, researchers have mapped m^6^A methylation profiles under drought and found clear differences between sensitive and tolerant lines (Miao et al., 2020). A similar pattern emerges in switchgrass: long-term salt stress tolerance is associated with dynamic changes in m^6^A methylation (Liu H. et al., 2024). Studies in wheat examining the interplay between m^6^A modification and virus infection have revealed variety-specific patterns in virus-host interaction pathways (Zhang et al., 2021). Future work needs to use multi-omics approaches to systematically understand how m^6^A regulation behaves under combined stresses and how it links with the plant’s broader adaptive networks.

Furthermore, field-level validation of m^6^A-based breeding strategies remains relatively limited. For most m^6^A regulatory factors reported to date, the available evidence comes primarily from controlled greenhouse or growth chamber environments, where variables are tightly regulated. Whether the stress-tolerant phenotypes observed under these conditions can be reproduced in more variable field settings—where plants are exposed to fluctuating temperatures, changing soil moisture, and complex biotic interactions—has yet to be adequately demonstrated for the majority of these factors. Bridging this gap will require multi-environment field trials across different years and locations, which are both time-consuming and resource-intensive. Genotype-by-environment interactions, which are known to complicate conventional breeding efforts, are likely to be equally relevant for epigenetic traits and warrant further investigation in the context of m^6^A-mediated stress tolerance (Johannes et al., 2009).

Given that most m^6^A-based strategies remain at the proof-of-concept stage and still require multi-environment field trials, regulatory approval, and public acceptance, the first m^6^A-edited stress-resilient varieties are unlikely to reach farmers’ fields within the next 5–10 years; the earliest applications are most plausible in vegetatively propagated or high-value horticultural crops, where transformation systems are more mature and regulatory pathways are more established.

In addition, most studies so far have focused on single m^6^A factors or individual target genes. However, m^6^A modification affects stress resistance more broadly by modulating the stability and translation efficiency of the entire transcriptome. Therefore, we also need to move from single-gene studies to network-level regulation. A few examples already hint at this complexity: in Arabidopsis, MTA influences mRNA under drought in both m^6^A-dependent and m^6^A-independent ways (Ganguly et al., 2025). In rice, polysome-bound mRNA analysis under drought showed that m^6^A modification affects translation efficiency. In maize, natural variation in m^6^A methylation has been linked to translation (**Figure 4**).

**Figure 4:**
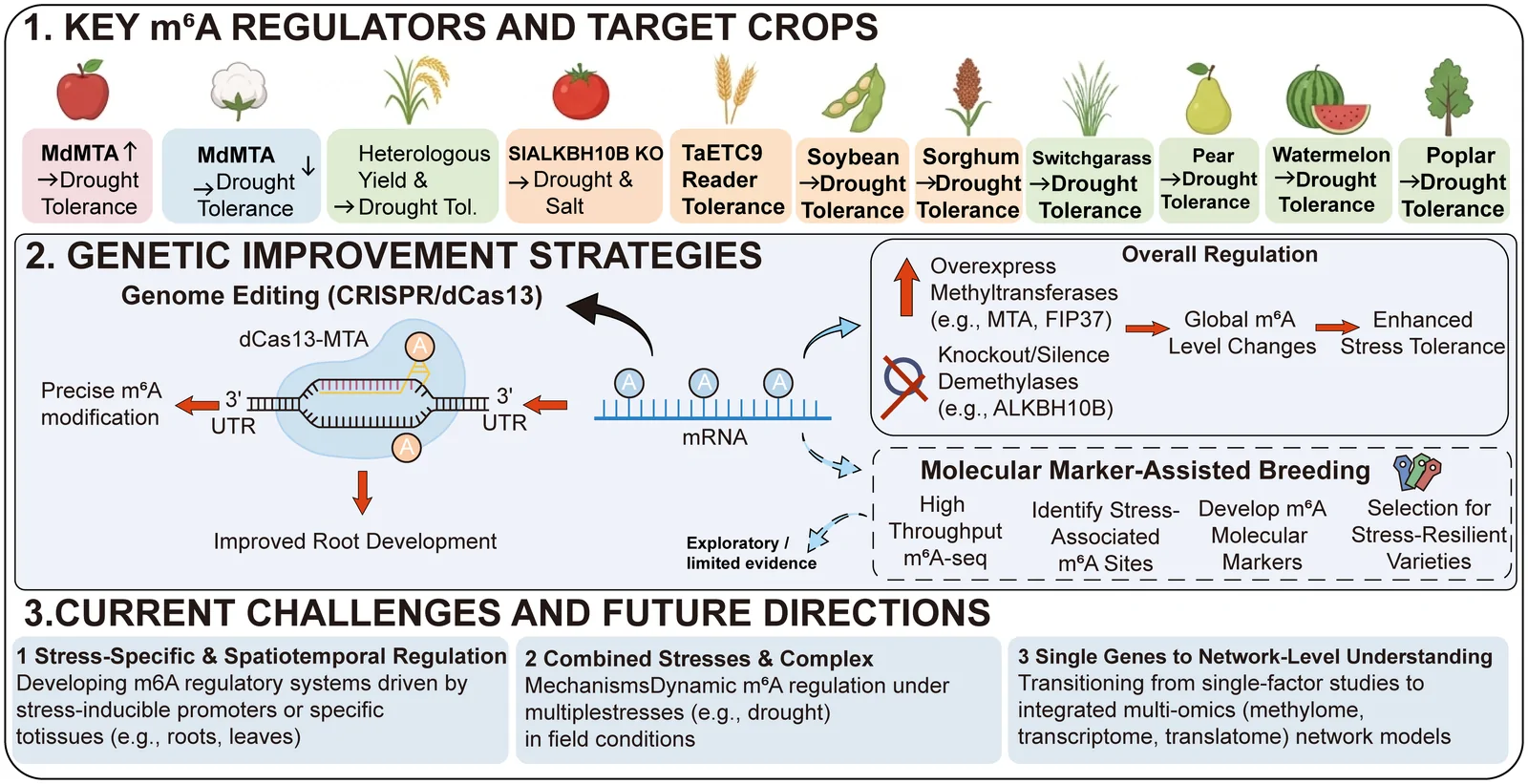
Application potential, genetic improvement strategies, and future perspectives of m^6^A regulation in crop stress-resistance breeding. Application potential, genetic improvement strategies, and future perspectives of m^6^A regulation in crop stress-resistance breeding. Key regulators with demonstrated stress tolerance effects are listed (left). The strategies include targeted epigenome editing via CRISPR/dCas13 (solid boxes and arrows; supported by experimental evidence), global manipulation through overexpression or knockout (solid boxes and arrows; supported by experimental evidence), and marker-assisted selection (dashed boxes and arrows; limited evidence, currently at an exploratory stage). Current challenges include developing spatiotemporal control, understanding responses under combined stresses, and transitioning from single-gene to network-level integration (right).

## 6 Conclusions and perspectives

### 6.1 Major advances

Recent work has greatly expanded our understanding of m^6^A methylation in plant stress responses. Studies in Arabidopsis and various crop species have identified key regulatory components of the writer–eraser–reader network (Parker et al., 2020; Kong et al., 2023; Cai J. et al., 2024; Cai Z. et al., 2024; Li et al., 2025; Zhang C. et al., 2025). These findings have established that m^6^A modification modulates mRNA stability, translation efficiency, splicing, and polyadenylation under stress conditions. Functional validation of specific m^6^A regulators (e.g., *FIONA1*, *ECT8*, *ClMTB*, and *CmALKBH8*) has provided genetic evidence linking m^6^A modification to salt tolerance, drought resistance, and fruit ripening (He et al., 2021; Cai J. et al., 2024; Cai Z. et al., 2024; Li et al., 2025). However, the downstream targets and phenotypic outcomes of m^6^A regulators are not uniform across species: *ALKBH10B* regulates flowering in Arabidopsis, whereas its orthologs primarily mediate stress tolerance in cotton and tomato; similarly, loss of *MTA* causes embryonic lethality in Arabidopsis, whereas *MTA* overexpression enhances drought tolerance in apple (Zhong et al., 2008; Hou et al., 2022; Li B. et al., 2023; Shen et al., 2023). In addition, m^6^A has been shown to regulate systemic stress signaling through long-distance transport of specific mRNAs (Parker et al., 2020). Together, these studies demonstrate that, although the core m^6^A machinery is broadly conserved, its regulatory functions have diverged considerably among species.

### 6.2 Existing challenges and future perspectives

Several challenges remain in applying m^6^A methylation to breeding. First, single-base m^6^A mapping remains technically challenging in most crops, although nanopore direct RNA sequencing offers promising resolution (Zhao et al., 2019; Ferraz et al., 2023; Kong et al., 2023). Second, the functions of m^6^A regulatory components across diverse species and tissues are not fully understood; consequently, findings from model plants cannot be directly extrapolated to crops without experimental validation (Yu et al., 2025a). Third, direct experimental evidence linking specific m^6^A methylation events to downstream gene expression and trait outcomes is often lacking (Mansoor et al., 2025). Fourth, the stability and heritability of stress-induced m^6^A changes under field conditions involving multiple simultaneous stresses require further investigation (Liang et al., 2023).

Looking ahead, several research areas will become important. At the fundamental level, efforts should focus on developing high-resolution detection tools and on identifying key m^6^A regulators in various crops. Recent advances in single-cell and spatial transcriptomics offer new opportunities for dissecting cell-type-specific m^6^A regulation (Zhang B. et al., 2024; Yu et al., 2025b). Although current m^6^A profiling methods (e.g., meRIP-seq) average signals across heterogeneous cell populations (Iqbal et al., 2020), integrating single-cell or spatial approaches with m^6^A profiling could reveal cell-type-specific methylation dynamics (Feng et al., 2023; Barmukh et al., 2025). Integrating multi-omics datasets—transcriptomic, methylomic, and phenotypic—will be crucial for reconstructing regulatory networks and establishing causal links between m^6^A events and agronomic traits (Liang et al., 2023). At the applied level, developing CRISPR/Cas-based tools for targeted m^6^A editing offers new opportunities for precision breeding, although efficient genetic transformation systems remain a bottleneck in many crops. Ultimately, combining epitranscriptomic knowledge with molecular breeding strategies may contribute to the development of crop varieties with improved stress resilience (**Figure 5**).

**Figure 5:**
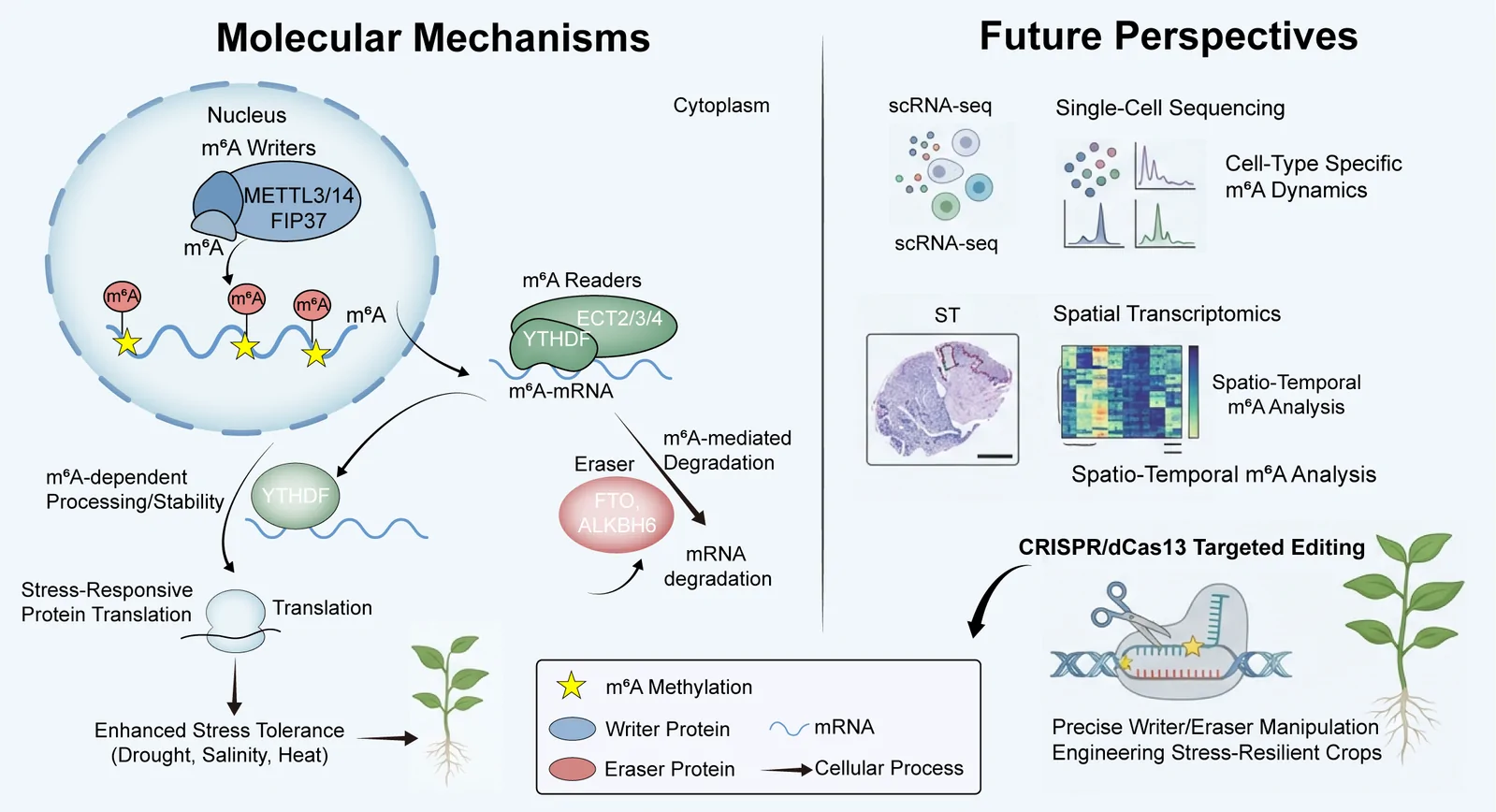
Molecular mechanisms and future perspectives of m^6^A modification in plant stress responses. The left panel illustrates the m^6^A regulatory pathway in plant cells. In the nucleus, m^6^A writers (e.g., METTL3/14, FIP37) install modifications on target mRNAs. After export to the cytoplasm, these methylated mRNAs are recognized by m^6^A readers such as ECT2/3/4 and other YTHDF proteins, which are involved in modulating mRNA processing and stability, or facilitating the translation of stress-responsive proteins. Alternatively, the m^6^A marks can be removed by erasers (e.g., FTO, ALKBH6), leading to mRNA degradation. The right panel highlights future directions. Emerging approaches like single-cell RNA sequencing (scRNA-seq) and spatial transcriptomics (ST) are expected to reveal cell-type-specific m^6^A dynamics and spatiotemporal patterns. Furthermore, CRISPR/dCas13-based targeted editing offers a potential avenue for manipulating m^6^A writers or erasers to engineer stress-resilient crops.
